# A Smart Epoxy Composite Based on Phase Change Microcapsules: Preparation, Microstructure, Thermal and Dynamic Mechanical Performances

**DOI:** 10.3390/molecules24050916

**Published:** 2019-03-06

**Authors:** Qinghong Hu, Yan Chen, Jiaoling Hong, Shan Jin, Guangjin Zou, Ling Chen, Da-Zhu Chen

**Affiliations:** 1 SDCIC Construction Group Co., Ltd., Shenzhen 518038, China; dianfan520@163.com (Q.H.); ecomaterial@163.com (S.J.); qiaoyudezhanghao@gmail.com (G.Z.); 2 Shenzhen Key Laboratory of Polymer Science and Technology, College of Materials Science and Engineering, Shenzhen University, Shenzhen 518055, China; cocobay2008@hotmail.com (Y.C.); 2172341455@email.szu.edu.cn (J.H.); 3 Department of Industrial and Systems Engineering, The Hong Kong Polytechnic University, Hung Hom, Kowloon, Hong Kong, China

**Keywords:** phase change materials, microcapsules, epoxy composites, dynamic mechanical properties, thermal regulation

## Abstract

Microencapsulated phase change materials (MicroPCMs)-incorporated in epoxy composites have drawn increasing interest due to their promising application potential in the fields of thermal energy storage and temperature regulation. However, the study on the effect of MicroPCMs on their microstructure, thermal and viscoelastic properties is quite limited. Herein, a new type of smart epoxy composite incorporated with polyurea (PU)-shelled MicroPCMs was fabricated via solution casting method. Field emission-scanning electron microscope (FE-SEM) images revealed that the MicroPCMs were uniformly distributed in the epoxy matrix. The thermal stabilities, conductivities, phase change properties, and dynamic mechanical behaviors of the composite were studied by differential scanning calorimetry (DSC), thermogravimetric analysis (TGA), dynamic mechanical analysis (DMA), thermal constant analyzer and infrared thermography. The results suggested that the heat storage ability of the composites was improved by increasing the MicroPCMs content. The thermal stability of MicroPCMs was found to be enhanced after incorporation into the matrix, and the MicroPCMs-incorporated epoxy composites showed a good thermal cycling reliability. Moreover, the incorporation of MicroPCMs reduced the composites’ storage modulus but increased the glass transition temperature (*T*_g_) as a result of their restriction to the chain motion of epoxy resin. Besides, a less marked heating effect for the composite was explored through infrared thermography analysis, demonstrating the good prospect for temperature regulation application.

## 1. Introduction

Phase change materials (PCMs) have the ability of thermal energy storage and thermo-regulation during phase transition process by absorbing and releasing of latent heat over a narrow temperature range [1,2]. For PCMs undergo the solid-liquid phase transition process, encapsulation technology is usually required to produce microencapsulated PCMs (MicroPCMs) before incorporating into the final system. With a core-shell structure and a large heat transfer surface area, MicroPCMs can improve the thermal stability, thermal conductivity of the conventional PCMs, and avoid the super-cooling problem and PCM leakage [3,4,5,6].

Design and development of MicroPCMs with high energy storage capacity and appropriate phase change temperature range for different applications is one of the research focuses in the field of temperature regulation and energy storage [7,8]. Various types of natural or synthetic materials have been developed and used to fabricate MicroPCMs. Choosing of core and shell materials plays a vital role for the design of MicroPCMs. Paraffin is one of the widely used commercial organic PCMs, showing great cyclic stability [9]. The melting temperature of the paraffin can be altered by changing the molecular weight and chain length [10]. Of this kind, *n*-octadecane shows a mild phase change temperature of around 28 °C and a high latent heat of 241.2 J g^−1^ [11]. In this regard, *n*-octadecane has been encapsulated for use with different shell materials, such as polystyrene [12], polymethyl methacrylate [13], melamine-formaldehyde (MF) resin [14], urea-formaldehyde (UF) resin [15], silicon dioxide [16] and calcium carbonate [17], to obtain desirable thermal and structural properties. 

To realize the practical application in temperature regulation and thermal energy storage, MicroPCMs generally need to be integrated into variety of matrices, such as fibers and fabrics, building construction materials, and mobile phone cooling materials [7,18,19,20]. Among the supporting materials, epoxy resin has recently aroused increasing research interests due to its good mechanical properties, thermal stability, solvent resistance and easy preparation [21]. Su et al. [22,23,24,25] fabricated the MF-shelled MicroPCMs-filled epoxy composites and systematically researched the thermal conductivity and interface debonding phenomenon between the shell and matrix. The thermal transmission ability of the MicroPCMs/epoxy composites was improved with smaller size MicroPCMs containing the same amount of PCM [22]. Interface debonding was generated due to the expansion coefficient mismatch between the methanol–melamine–formaldehyde (MMF) shells and epoxy after a violent thermal shock treatment [23,24]. Moreover, increasing the thermal conductive speed and repeated times of thermal absorbing-releasing process would lead to a larger debonding stress [25]. It was also reported that large particle size of MicroPCMs and interface area would give more structure defects and have negative impacts on the mechanical properties of the MicroPCMs/epoxy composites [26]. However, to the best of our knowledge, few researches have been devoted to the basic understanding of viscoelastic properties, thermal stability and temperature regulation capacity of these smart MicroPCMs/matrix composites.

In this work, PU shelled *n*-octadecane MicroPCMs with a large latent heat were fabricated by facile interfacial polymerization between isophorone diisocyanate (IPDI) and diethylenetriamine (DETA) in the oil-water interface. The as-prepared MicroPCMs-incorporated novel epoxy composites were fabricated and characterized in terms of morphology, thermal storage ability, thermal conductivity, viscoelastic properties and thermal stability. The effect of MicroPCMs filling ratio on these properties was well discussed. This study provided useful information for the design and fabrication of smart MicroPCMs/epoxy composites for thermal regulation use. 

## 2. Results and Discussion

### 2.1. Synthesis and Structure of PU-Shelled n-Octadecane MicroPCMs

**Figure 1:**
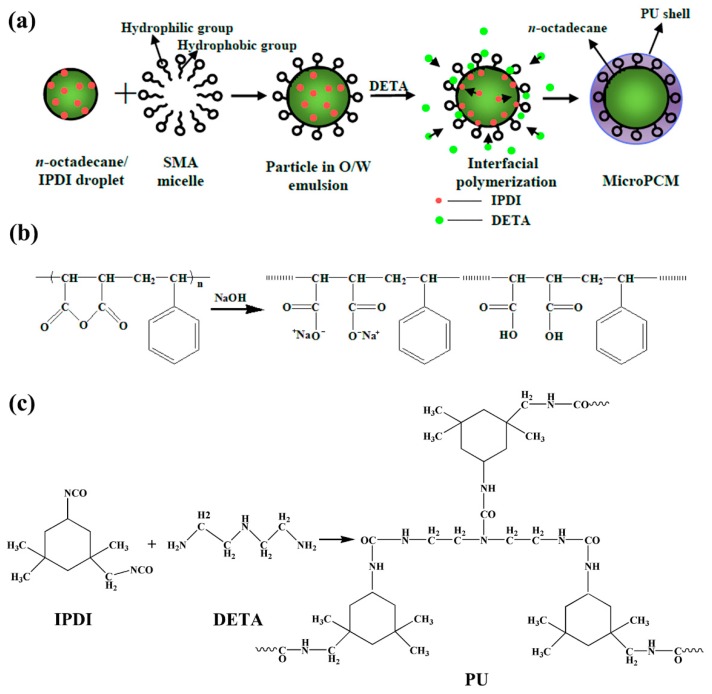
Schematic formation of the microencapsulated *n*-octadecane with the PU shell via interfacial polymerization. (**a**) Synthesis process of the microcapsules, (**b**) molecular structure of SMA and its hydrolyzed form, and (**c**) the polymerization reaction of PU shell.

**Figure 2:**
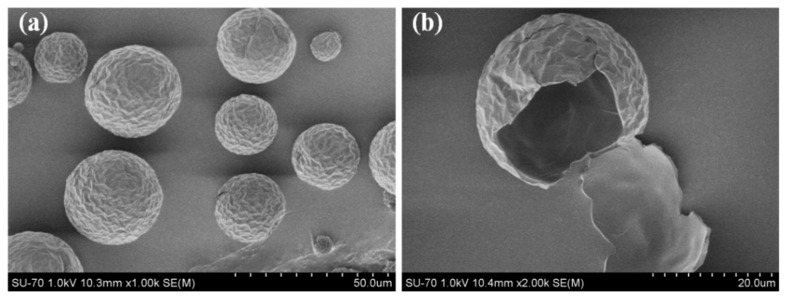
SEM images of (**a**) MicroPCMs, (**b**) the fracture surface of a MicroPCM.

**Figure 3:**
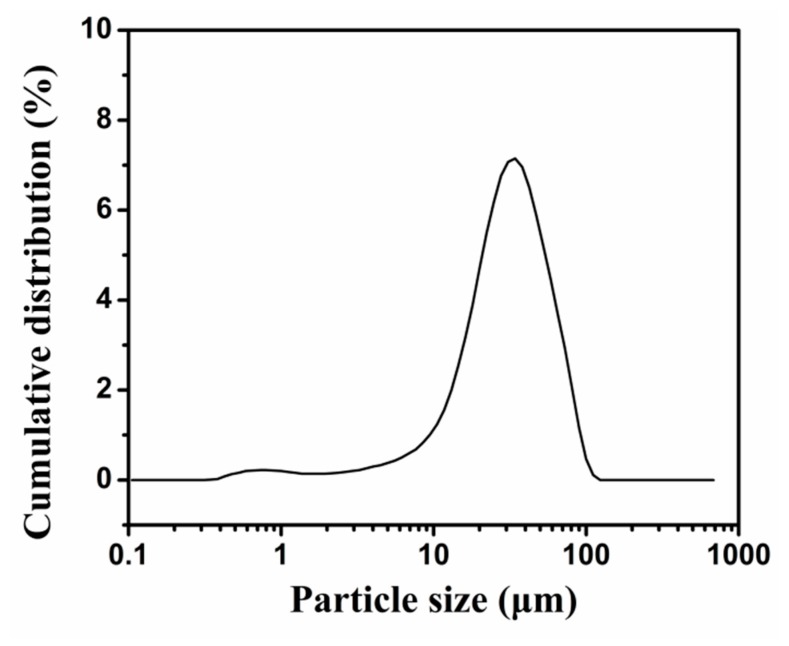
The particle size distributions of MicroPCMs determined using a laser particle size analyzer.

The schematic diagram of the formation of the microencapsulated *n*-octadecane with PU shell by interfacial polymerization is shown in Figure 1a. Firstly, the mixed *n*-octadecane and IPDI organic solution was dispersed in an aqueous solution consisting of SMA as an emulsifier, to produce an oil-in-water (O/W) emulsion. SMA is a water-soluble polyelectrolyte copolymerized with styrene and maleic anhydride monomers. The anhydride groups of SMA could be hydrolyzed in alkaline water solution and form carboxy groups (-COOH) and carboxylate ions as the hydrophilic groups (Figure 1b). The hydrolyzed SMA plays the roles of dispersant and anionic polyelectrolyte during the formation of microcapsules. The hydrophilic groups of SMA, alternatively arranging along its hydrophobic backbone chains, are associated with the water molecules and trimly cover the surface of the *n*-octadecane/IPDI oil droplets with hydrophobic groups, oriented into oil droplet and hydrophilic groups in contact with water phase, resulting in stable micelles [111]. Subsequently, DETA was added into the emulsion to react with IPDI. The formation of PU shell occurred at the interface between the *n*-octadecane droplets containing IPDI monomer and aqueous solution containing DETA monomer. At the oil-water interface, IPDI might take a side reaction with water molecules to form –NH_2_ groups and reacted with other IPDI molecules. However, due to the faster reaction rate, the isocyanate groups of IPDI would mainly directly react with the amine groups of DETA to produce urea linkage (Figure 1c). Through these reactions, a urea-linked polymeric shell was formed and *n*-octadecane was encapsulated in it. In this study, the reaction temperature was maintained to be 60 °C. To achieve a good dispersion of the synthesized MicroPCMs and a modest reaction speed, diluted DETA was added into the emulsion dropwise with a stirring speed of 500 rpm. The FE-SEM images of MicroPCMs and a fractured microcapsule are shown in Figure 2. In Figure 2a, it can be clearly observed that the MicroPCMs are in a golf ball-like shape and have a compact shell surface with some folds on it. The folds are probably formed due to the shrinkage of the core material during the drying process. The thickness of the PU shell is determined to be about 1 μm from the fracture surface of MicroPCMs (Figure 2b). The size distributions of the microcapsules were tested by a BT-9300ST laser particle size analyzer. As shown in Figure 3, the mean particle size of the prepared MicroPCMs is 34 μm. 

**Figure 4:**
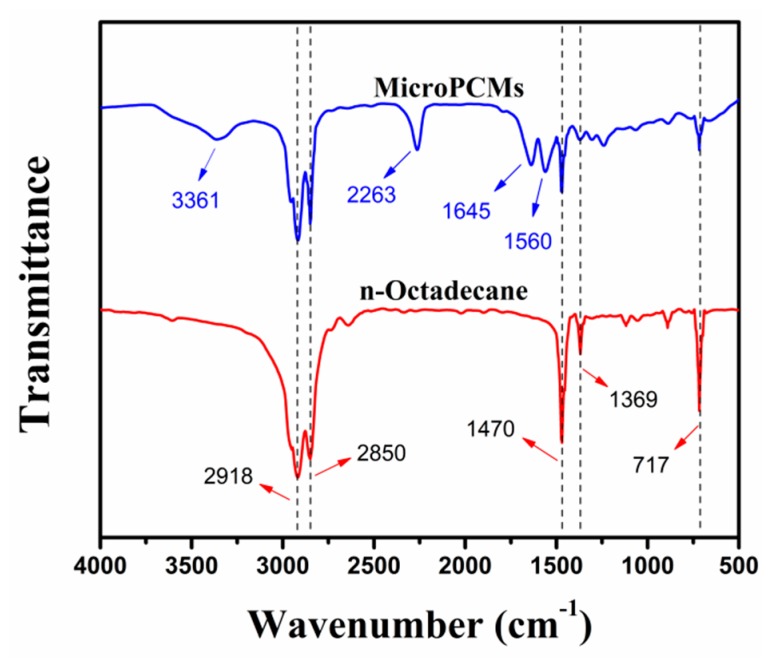
FTIR spectra of *n*-octadecane and MicroPCMs.

Figure 4 shows the Fourier transform infrared (FTIR) spectra of *n*-octadecane and MicroPCMs. In the spectrum of *n*-octadecane, strong absorbance at 2918 cm^−1^ and 2850 cm^−1^ correspond to the aliphatic C-H stretching vibrations of –CH_2_ and –CH_3_, respectively. The peak at 1369 cm^−1^ are attributed to the bending vibration of −CH_3_, while the peaks at 1470 cm^−1^ and 717 cm^−1^ are associated with the in-plane bending vibration peaks of –CH_2_. 

In the spectrum of MicroPCMs, the absorption bands at 3361 cm^−1^ and 1560 cm^−1^ are assigned to the hydrogen-bonded –NH stretching vibration. The peak at 2263 cm^−1^ is associated with the rest unreacted –NCO group in the core material. The urea carbonyl stretching vibration associated with hydrogen bonding is found at 1645 cm^−1^ [27]. Besides, all the characteristic peaks of *n*-octadecane can be observed in the MicroPCMs spectrum, confirming the successful encapsulation of *n*-octadecane with PU through the interfacial copolymerization process.

### 2.2. Microstructure of MicroPCM/Epoxy Composites

**Figure 5:**
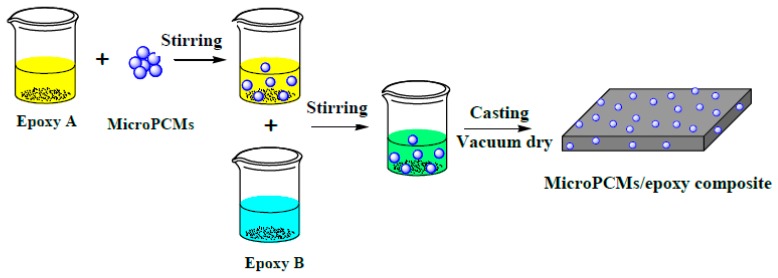
Schematic diagram of the fabrication of MicroPCMs/epoxy composites by a casting method.

The MicroPCMs/epoxy composites were fabricated through a solution casting method which includes the procedures of blending and curing as shown in Figure 5 (see details in the experimental section).

**Figure 6:**
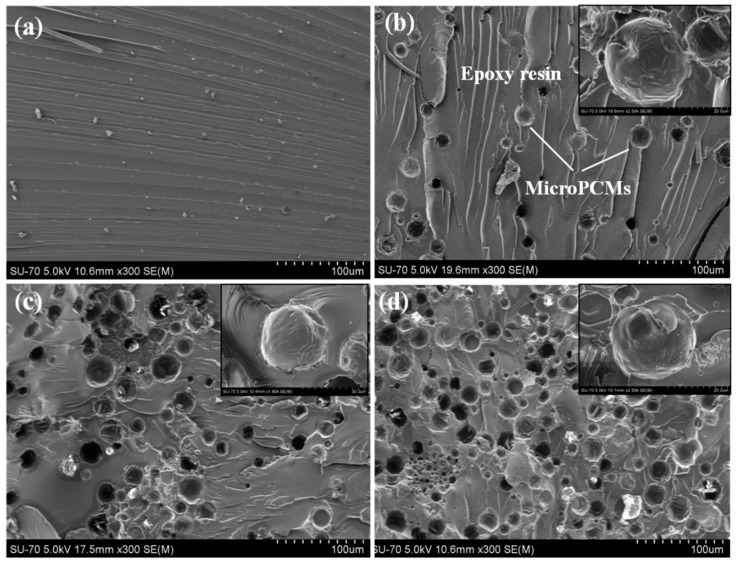
SEM images of MicroPCMs/epoxy composites with MicroPCMs contents of (**a**) 0 wt%, (**b**) 5 wt%, (**c**) 10 wt%, (**d**) 20 wt%, inserted with images with high magnification.

The FE-SEM images of the cross-section of MicroPCMs/epoxy composites are shown in Figure 6. It is found that in the MicroPCMs-filled epoxy composite, the numbers of the MicroPCMs and spherical holes increase with the increasing content of MicroPCM. The MicroPCMs are uniformly distributed in the epoxy matrix without significant agglomeration and breakage. A good interfacial bonding between the MicroPCMs and the epoxy matrix is achieved, as shown in the SEM images with high magnification (inserted images). The coarse surface of MicroPCMs with abundant folds (Figure 2) provides substantial points for epoxy resin attachment and fixation according to the embedding mode, benefiting the interfacial bonding between microcapsules and epoxy matrix. Similar results were also observed in the MicroPCMs-incorporated cement composites [4]. 

### 2.3. Phase Change Properties

**Figure 7:**
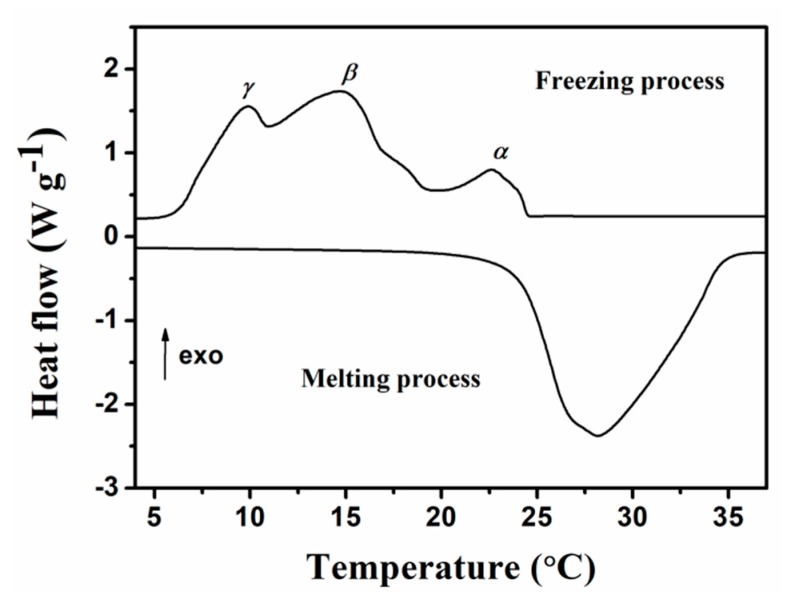
DSC curve of MicroPCMs.

Thermal properties of the microencapsulated *n*-octadecanes were determined using a TA Q200 differential scanning calorimeter (DSC) in a temperature range of −10 to 50 °C at the heating/cooling rate of 5 °C min^−1^. The DSC curve and phase change performances of the MicroPCMs are presented in Figure 7 and Table 1. The melting process shows a single peak with a onset temperature (*T*_m,s_) of 24.10 °C and an end temperature (*T*_m,e_) of 34.70 °C. The melting point (*T*_m_), determined from the peak temperature, is 28.14 °C. Owing to the confinement of phase change behavior of *n*-octadecane in the small space of the microcapsules [28], three crystallization peaks (nominated as α-, β- and γ-peak) appear in the cooling DSC curve, which are related to the heterogeneously nucleated liquid-rotator phase transition, homogeneous nucleated liquid-crystal phase transition, and rotator-crystal transition. Similar results were shown in the microencapsulated *n*-octadecanes with the shell of melamine-formaldehyde resin [28,29] or silica shell [16]. However, in some other literatures on microcapsulated *n*-octadecanes, only two crystallization peaks (α, β) were observed in the DSC curve [17,27,30]. Generally, the specific crystallization of *n*-octadecane within microcapsules is complicated, which is affected by the confined geometry, shell roughness, microcapsule size [29], and as well as the purity of the commercial PCM. The crystallization temperatures of the as-prepared MicroPCMs, *T*_α_, *T*_β_, *T*_γ_ (related to α-, β- and γ-crystals) are 22.58 °C, 14.67 °C and 9.90 °C, respectively. The latent heats of the MicroPCMs are measured to be 182.4 J g^−1^ for the fusion process (Δ*H*_m_) and 183.2 J g^−1^ for the crystallization process (Δ*H*_f_), respectively. 

**Figure 8:**
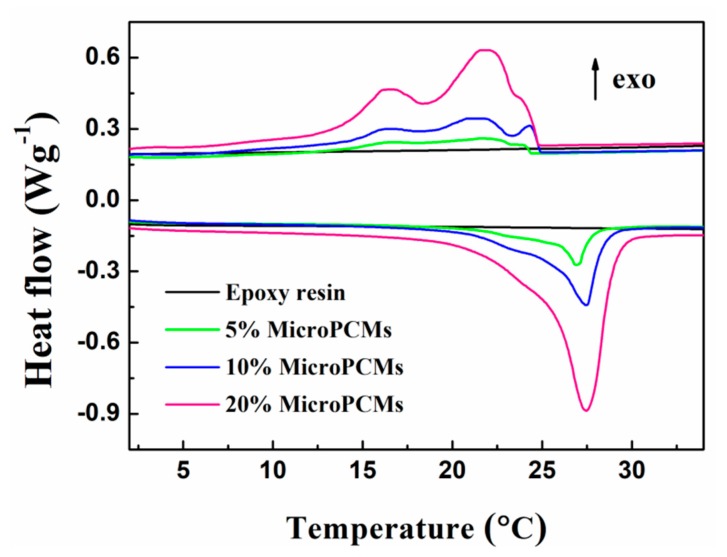
DSC curves of pure epoxy resin and MicroPCMs/epoxy composites with different MicroPCM contents.

The DSC thermograms of pure epoxy resin and MicroPCMs/epoxy composites with different filling ratios are shown in Figure 8. For the unfilled epoxy resin, no endothermic or exothermic peaks can be found in the DSC thermogram, which implies that the thermal effects of the composites are mainly attributed to the incorporated MicroPCMs. The phase change properties obtained from DSC analysis are listed in Table 1. The whole melting process of the epoxy composites containing 5 wt%, 10 wt% and 20 wt% MicroPCMs occurs in the temperature range of approximately 15–32 °C, and no significant difference of *T*_m_ can be observed. In the freezing process, the solidification of PCMs incorporated in the composites falls in the temperature scope ranging from 7 °C to 25 °C. Besides the main crystallization peak (β), α- and γ- crystallization peaks can still be observed in the DSC curves for the composites. It is clearly observed that both the Δ*H*_m_ and Δ*H*_f_ increase with increasing MicroPCM content in the composite. In the case of the epoxy composite containing 20 wt% MicroPCMs, the value of Δ*H*_m_ reaches to 33.24 J g^−1^, 4.4 times of that of the composite with 5 wt% MicroPCM filling ratio. Therefore, the incorporation of MicroPCMs endows the epoxy with energy storage ability which can be well tuned by varying the filling amounts. 

### 2.4. Thermal Stability

**Figure 9:**
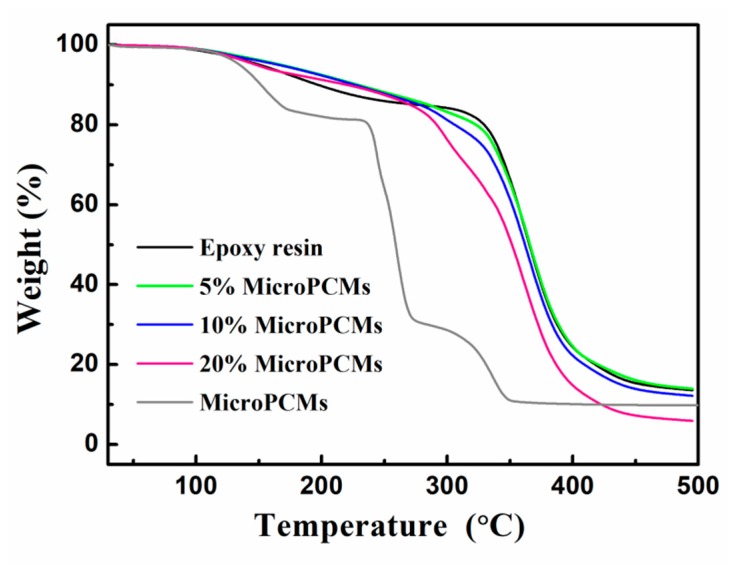
TGA thermograms of MicroPCMs, pure epoxy resin and MicroPCMs/epoxy composites with different MicroPCM contents.

Figure 9 presents the TGA thermograms of MicroPCMs, pure epoxy resin and MicroPCMs-filled composites. The degradation of MicroPCMs took place in the temperature range from 100 to 360 °C, and included three sections. The first two parts occurred before 270 °C were attributed to the evaporation and decomposition of the core material, while the third part corresponded to the thermal decomposition of PU shell of MicroPCMs. It can be observed that the weight loss rate was substantially decreased when the MicroPCMs were incorporated into epoxy matrix. To achieve a weight loss of 10%, for instance, the MicroPCMs must be heated to 197.58 °C, while for the epoxy composite filled with 5 wt%, 10 wt% and 20 wt% MicroPCMs, the decomposition was delayed to 229.59 °C, 227.36 °C, and 222.15 °C, respectively. The results suggested that the epoxy matrix had a certain positive effect of improving the thermal stability of MicroPCMs. 

**Figure 10:**
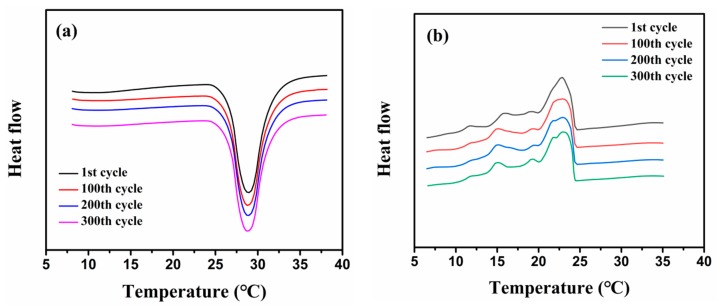
DSC curves of the MicroPCMs-filled epoxy composite after experiencing 1, 100, 200, and 300 heating/cooling cycles during the (**a**) melting and (**b**) freezing processes.

Figure 10 presents the DSC curves of the epoxy composite containing 20 wt% MicroPCMs after 1, 100, 200, and 300 heating/cooling cycles. The composite from the first cycle to the 100th, 200th, and 300th cycles shows good coincident melting and freezing peaks, which suggests the phase change temperatures are quite close and there is almost no change of the latent heats in the composite after experiencing 300 heating/cooling cycles. It is evident that the MicroPCMs-filled epoxy composite has behaved a good thermal cycling reliability.

### 2.5. Dynamic Mechanical Properties

**Figure 11:**
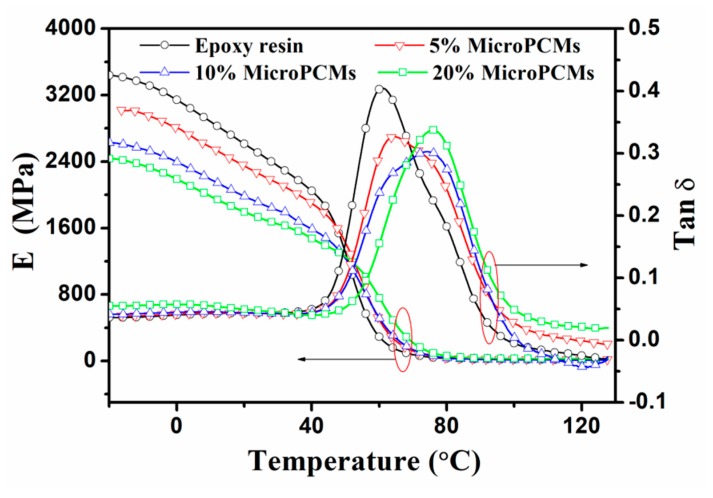
DMA thermograms of pure epoxy resin and MicroPCMs/epoxy composites with various MicroPCM contents.

Plots of the storage modulus (*E′*) and tangent of loss angle (tan *δ*) vs. temperature for pure epoxy resin and MicroPCMs/epoxy composites with different MicroPCMs filling contents are shown in Figure 11. Epoxy resin kept its intrinsic viscoelastic characteristic with the incorporation of MicroPCMs. The composites showed a high *E′* value with only the change of bond length and bond angle at low temperatures. As the temperature rose, the segmental motion of molecular chains became easier, the glass transition occurred and the modulus decreased gradually. The softening of MicroPCMs with increasing temperature is another reason for the reduction of modulus. With further increased temperature, the macromolecular chains began to move and underwent a viscous flow transition. It can be seen that the *E′* of composite systems decreased monotonously with the increase of MicroPCMs content. Similar results were observed in the MicroPCMs-filled biopolymer composite [31] and self-healing microcapsules-filled visible light cured polymer composite [32]. It was quite different from the reinforcing effect discovered in polymeric composites incorporated with rigid inorganic particles [33,34,35]. The reason is that MicroPCMs generally behave a lower strength compared to the epoxy resin matrix, and as a result, the more the filling content of MicroPCMs, the lower the rigidity of the composites. Nevertheless, the epoxy composites can still maintain a high storage modulus even if the MicroPCMs concentration was increased to 20 wt%. For instance, the storage moduli of the composite containing 20 wt% MicroPCMs reached to the level as high as 2.36 GPa at −10 °C and 1.79 GPa at 20 °C. Moreover, the dependence of *E′* on MicroPCMs filling fractions suggested that to design energy storage composites materials, we should not only consider the increase of phase change enthalpy, but also take account of mechanical properties and thermal storage capacity for the practical application environment. The glass transition temperatures (*T*_g_) of bare epoxy resin was 59.77 °C, determined from the peak temperature of the tan *δ*-*T* curve. After incorporation of 5 wt%, 10 wt% and 20 wt% MicroPCMs, the *T*_g_ values for the composites were increased to 65.77 °C, 71.84 °C and 74.25 °C, respectively, indicating that the presence of MicroPCMs restricted the segmental movement of epoxy resin.

### 2.6. Evaluation of Temperature Regulation Capacity

Thermal conductivity is one of the essential properties to estimate the thermal-responsive rate of MicroPCMs-filled composite for thermal regulation application. The thermal conductivity of the unfilled epoxy resin was measured to be 0.34 W m^−1^ K^−1^, and became 0.30 W m^−1^ K^−1^, 0.29 W m^−1^ K^−1^ and 0.27 W m^−1^ K^−1^, respectively, when 5 wt%, 10 wt% and 20 wt% of MicroPCMs were incorporated. The slight descending tendency of the thermal conductivity with increasing the MicroPCMs content also suggested that a balance should be achieved between the thermal storage capacity and the thermal transfer rate by adjusting the content of MicroPCMs. 

**Figure 12:**
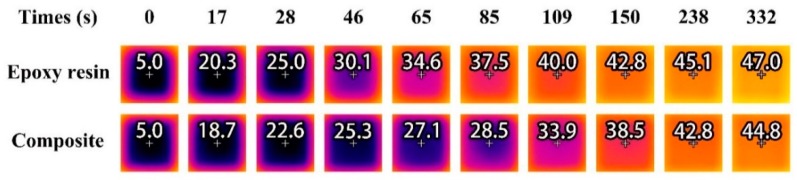
Thermal images of the square sheets of pure epoxy resin and the epoxy composite filled with 20 wt% MicroPCMs. The temperatures at the white cross were marked on the thermal images.

Figure 12 presents the infrared thermography of the surface temperature distribution of the bare epoxy sheet and the composite sheet filled with 20 wt% MicroPCMs taken at various times. It can be seen that, during the initial heating stage from the start to 28 s when the temperature change depends only on the sensible heat [4], the surface temperature of the composite sheet was found to behave a slight decrease of less than 2.5 °C, compared to that of epoxy sheet without MicroPCMs, due to the relatively lower thermal conductivity of the composite as mentioned above. After that, the temperature for the composite sheet was found to be 4.8 °C, 7.5 °C and 9.0 °C lower than that of the raw epoxy sample after 46 s, 65 s and 85 s, and 410 s, respectively. During this period, the involved phase change material began to melt and absorb the ambient heat, which effectively suppress the temperature rising on the composite surface. Thereafter, the phase change process finished and only the sensible heat took effects, as a result, the descending degree of surface temperature between the samples with and without MicroPCMs became small. The less marked heating effect (so-called “delay effect” [20]) for the composite specimen indicates that the incorporation of MicroPCMs could effectively regulate ambient temperature by absorbing/releasing the latent heat. 

## 3. Experimental

### 3.1. Materials

*n*-Octadecane with 98% purity (Aladdin Chemistry Co. Ltd., Shanghai, China) was used as the core material. Isophorone diisocyanate (IPDI) and diethylene triamine (DETA), purchased from Alfa Aesar Company (Shanghai, China), were used as the reacting monomers to form the microcapsule shell. Styrene-maleic anhydride copolymer (SMA) with the weight-average molecular weight of 350,000, purchased from Beijing THK Sci. Co. Ltd. (Beijing, China), was used as the emulsifier. AB epoxy adhesive, supplied by Hangzhou Five Port Adhesive Co. Ltd. (Hangzhou, China), acted as the polymer matrix of the composite. Sodium hydroxide (NaOH) and sodium chloride (NaCl) were supplied by Tianjin Fu Chen Chemical Reagent Factory (Tianjin, China).

### 3.2. Preparation of PU-Shelled n-Octadecane MicroPCMs

The PU microencapsulation of *n*-octadecane was synthesized by the technique of interfacial polymerization (Figure 1). The fabrication procedure of PU/*n*-octadecane MicroPCMs included four steps: (i) preparation of aqueous solution and oil solution, (ii) mixing and emulsification, (iii) microencapsulation, and (iv) post-treatment. To prepare the aqueous solution, 20 g SMA was dissolved in 250 mL of 3 wt% NaOH water solution at 90 °C under stirring at 500 rpm for 3 h. The oil solution was prepared by mixing 7.0 g *n*-octadecane and 3.0 g IPDI under magnetic stirring. The aqueous and the oil solution were mixed and agitated at a speed of 5000 rpm for 20 min at 60 °C for the emulsification. Subsequently, stoichiometric DETA was added dropwise into the above oil/water (O/W) emulsion to initiate the interfacial polymerization. After 3-h reaction at 60 °C and 500 rpm, a MicroPCM suspension was obtained. Post-treatments such as filtration, washing (60 °C, 3 times), and freeze-drying (−77 °C, 24 h) were carried out to filter out the MicroPCMs. 

### 3.3. Fabrication of PU-Shelled n-Octadecane MicroPCMs Filled Epoxy Composites

PU-shelled *n*-octadecane MicroPCMs filled epoxy composites were fabricated by a casting method, as shown in Figure 5. Epoxy adhesive part A with the amount of 3.0 g was mixed with the predetermined amount of MicroPCMs by vigorously stirring, after which 1.0 g epoxy adhesive part B was also added. The mixture was under stirring until it was homogeneously mixed. Subsequently, the mixed MicroPCMs/epoxy A/epoxy B was poured into moulds with different shapes and allowed to solidify. In order to eliminate bubbles in the mixture, vacuum was applied at room temperature. The epoxy composites filled with various amounts of MicroPCMs were fabricated after 3 h vacuum dry at 50 °C. In this study, 5 wt%, 10 wt%, and 20 wt% of MicroPCMs were incorporated into the epoxy composites and denoted by 5%MicroPCMs, 10%MicroPCMs and 20%MicroPCMs, respectively. Unfilled epoxy was also fabricated as a control. 

### 3.4. Morphological Observation

The morphological observation on the MicroPCMs and the fractured surfaces of the MicroPCMs/epoxy composites were conducted by a field emission-scanning electron microscope (FE-SEM, SU-70, Hitachi, Tokyo, Japan). The composites were fractured after frozen in liquid nitrogen. Gold sputtered coating was performed prior to the observation. 

### 3.5. Size Measurement of MicroPCMs

The size distributions of microcapsules were tested by a BT-9300ST laser particle size analyzer (Dandong Bettersize Instruments Co. Ltd., Dandong, China) using deionized water as the dispersion medium.

### 3.6. FTIR Measurement

FTIR spectra of the GO-ODA and MEPCMs were measured using a PerkinElmer FTIR spectrometer (PerkinElmer Corporation, Norwalk, USA) in the range of 4000–600 cm^−1^ using the KBr sampling method.

### 3.7. Measurement of Phase Change Properties 

The phase change performances of MicroPCMs/epoxy composites were analyzed by a differential scanning calorimeter (DSC, TA Q-200, TA Instruments, Inc., New Castle, WA, USA) at a heating rate of 5 °C min^−1^ in the rage of 0–50 °C under a nitrogen atmosphere at a flow rate of 40 mL/min. Sample with a weight of 4–6 mg was used for each test. 

### 3.8. Thermal Stability Test

The thermal stability of MicroPCMs/epoxy composites was evaluated by a thermal gravimetric analyzer (TGA, TA Q-50, TA Instruments, Inc., New Castle, WA, USA) at a heating rate of 10 °C min^−1^ from room temperature to 500 °C under the protection of nitrogen. The sample weight of 5–10 mg was used for the test. To study the thermal stability of composites, a 300 DSC thermal cycling test of the composite containing 20 wt% MicroPCMs was conducted at a heating/cooling rate of 10 °C /min in the temperature range between 5 °C and 40 °C.

### 3.9. Measurement of Dynamic Mechanical Properties

A dynamic mechanical analyzer (DMA, TA Q-800, TA Instruments, Inc., New Castle, WA, USA) was used to investigate the dynamic mechanical properties of the composites, with dimensions of 35 mm × 12 mm × 3 mm, under a nitrogen atmosphere. The analysis was in a single cantilever mode at an oscillation amplitude of 15 μm and a frequency of 1 Hz. The heating rate was 3 °C min^−1^ and the temperature range was from −20 to 130 °C.

### 3.10. Thermal Conductivity Test 

The thermal conductivities of the composites were measured by a laser flash method. Thermal constants analyzer (TC-9000H, ULVAC-RIKO Inc., Yokohama, Japan) was adopted for the measurement. The sample size was Ф 10 mm × 2 mm. 

### 3.11. Infrared Thermography

**Figure 13:**
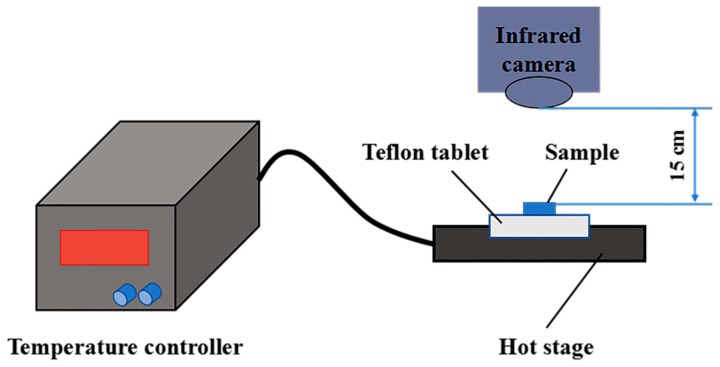
Schematic diagram of the test system with a hot stage to control the substrate temperature and an infrared camera (Fluke TiS65) to measure the surface temperature of the sample.

The thermo-regulating performance of the composite was evaluated with raw epoxy resin as the control using an infrared thermal imager (Fluke TiS65, Fluke Corporation, Washington DC, USA). The filling dosage in the measured composite sheets was 20 wt%. The sample size for the composite and control was 12 mm × 12 mm × 3 mm. All the samples were cooled to 3 °C prior to test and placed on a hot stage maintained at a constant temperature of 55 °C. The temperature of the hot stage was controlled with a temperature controller (K-3100, Guangzhou Ming Mei Technology Co., Ltd., Guangzhou, China). To prevent the sample temperature from rising too quickly, a cylindrical Teflon disc with 25 mm in diameter and 3 mm in thickness was placed between the hot plate and the test sample. The microscope lens was kept 15 cm from the upper surface of the sample during the test. The infrared thermal images were taken at different times until the temperature was increased to 50 °C, and analyzed using the SmartView 4.3 software. The whole test system was illustrated in Figure 13.

## 4. Conclusions

In this work, a novel epoxy composite incorporated with MicroPCMs using *n*-octadecane as the core and polyurea as the shell was successfully fabricated via solution casting method. The SEM results showed that the MicroPCMs with a spherical shape and a compact shell surface were uniformly distributed in the epoxy matrix with good interfacial bonding. The enhancement of thermal energy storage ability of the composites was achieved by increasing the filling fraction of MicroPCMs. In the case of the composite containing 20 wt% MicroPCMs, the melting latent heat reached to a level as high as 33.24 J g^−1^. TGA results suggested that the epoxy matrix could reduce the degradation rate of MicroPCMs and improved their thermal stability. The MicroPCMs-incorporated epoxy composites exhibited a good thermal cycling reliability. It was also found that the incorporation of MicroPCMs decreased the storage modulus to some degree owing to the relative soft feature of microcapsules, especially when the ambient temperature was situated in the phase transition zone of PCMs. Besides, the MicroPCMs restricted the movement of the polymer chain segments, resulting in a certain increase in the *T*_g_ values of the composites. Results from infrared thermography explored a less marked heating effect for the composite in comparison with pure epoxy resin, suggesting the promising application potential in the fields of thermal energy storage and temperature regulation. 

## Figures and Tables

**Table 1:** Phase change properties of the MicroPCMs/epoxy composites.

Sample	Melting Process				Freezing Process				
	*T*_m,s_ (°C)	*T*_m_ (°C)	*T*_m,e_ (°C)	Δ*H*_m_ (J g^−1^)	*T*_f,s_ (°C)	*T*_α_ (°C)	*T*_β_ (°C)	*T*_γ_ (°C)	Δ*H*_f_ (J g^−1^)
Epoxy resin MicroPCMs	24.10	28.14	34.70	182.40	24.51	22.58	14.67	9.90	183.20
5%MicroPCMs	14.71	27.41	31.36	7.55	24.17	24.08	23.46	17.57	7.58
10%MicroPCMs	14.85	27.31	31.13	15.77	24.54	24.25	23.46	15.30	14.92
20%MicroPCMs	16.50	27.95	31.60	33.24	24.64	24.02	23.79	16.01	32.27
